# Effect of Fibrin Concentration on the In Vitro Production of Dermo-Epidermal Equivalents

**DOI:** 10.3390/ijms22136746

**Published:** 2021-06-23

**Authors:** Andrés Montero, Cristina Quílez, Leticia Valencia, Paula Girón, José Luis Jorcano, Diego Velasco

**Affiliations:** 1 Department of Bioengineering and Aerospace Engineering, Universidad Carlos III de Madrid (UC3M), 28903 Madrid, Spain; anmonter@ing.uc3m.es (A.M.); cquilez@ing.uc3m.es (C.Q.); lvalenci@ing.uc3m.es (L.V.); 100329668@alumnos.uc3m.es (P.G.); 2 Instituto de Investigación Sanitaria Gregorio Marañón, 28007 Madrid, Spain

**Keywords:** fibrin hydrogels, plasma-derived fibrin hydrogel, bilayered in vitro skin substitutes, organotypic skin cultures, platelet-poor plasma (PPP), skin tissue engineering

## Abstract

Human plasma-derived bilayered skin substitutes were successfully used by our group to produce human-based in vitro skin models for toxicity, cosmetic, and pharmaceutical testing. However, mechanical weakness, which causes the plasma-derived fibrin matrices to contract significantly, led us to attempt to improve their stability. In this work, we studied whether an increase in fibrin concentration from 1.2 to 2.4 mg/mL (which is the useful fibrinogen concentration range that can be obtained from plasma) improves the matrix and, hence, the performance of the in vitro skin cultures. The results show that this increase in fibrin concentration indeed affected the mechanical properties by doubling the elastic moduli and the maximum load. A structural analysis indicated a decreased porosity for the 2.4 mg/mL hydrogels, which can help explain this mechanical behavior. The contraction was clearly reduced for the 2.4 mg/mL matrices, which also allowed for the growth and proliferation of primary fibroblasts and keratinocytes, although at a somewhat reduced rate compared to the 1.2 mg/mL gels. Finally, both concentrations of fibrin gave rise to organotypic skin cultures with a fully differentiated epidermis, although their lifespans were longer (25–35%) in cultures with more concentrated matrices, which improves their usefulness. These systems will allow the generation of much better in vitro skin models for the testing of drugs, cosmetics and chemicals, or even to “personalized” skin for the diagnosis or determination of the most effective treatment possible.

## 1. Introduction

In recent decades, the demand for in vitro skin substitutes that mimic human skin has increased both for in vitro toxicology testing and for clinical use, e.g., in wound coverage when dealing with large and/or severe wounds or burns [1,2,3,4,5]. In this way, the development and production of in vitro engineered skin substitutes has progressed towards more sophisticated cellular bilayered constructs containing dermal and epidermal components. The production of this type of 3D bilayered substitutes, either manually or through 3D bioprinting, generally involves the use of a 3D scaffold filled with fibroblasts (the dermal layer) and keratinocytes seeded on top (the epidermal layer) [6,7,8,9,10]. These matrices can be produced with either natural (e.g., alginate, collagen, fibrin, or hyaluronic acid) and/or synthetic products (e.g., polyethylene glycol, polycaprolactone, or polylactic acid [11,12,13,14]). Notably, human plasma-derived fibrin hydrogels are used as 3D scaffold in skin engineering [9,15,16,17]. Unlike renatured collagen or fibrin from commercial fibrinogen, the use of blood plasma as a source of human fibrin, together with human fibroblasts and keratinocytes from skin biopsies, allows for the production of autologous skin grafts. After grafting, natural collagen is efficiently produced by human fibroblasts and the concomitant remodeling of the scaffold generates a dermal extracellular matrix similar to that found in normal human skin [15,18]. Additionally, blood plasma composition [19] (plasma proteins, immunoglobulins, growth factors, enzymes, vitamins, and hormones) provides a more suitable 3D environment to promote the migration, proliferation, and differentiation of the cells in the wound bed [15,20,21]. With regard to platelet concentration, there are three groups: platelet-poor plasma (PPP), platelet-free plasma (PFP), and platelet-rich plasma (PRP). Usually, PPP and PFP are used indistinctly in the literature to denote blood plasma with a low platelet concentration [22,23]; however, there are experiments in which the absence of platelets is crucial. In these, PFP refers to blood plasma with no platelets [24,25]. By definition, PRP has at least 200–1000 × 103 platelets/μL suspended in plasma, which is attributed to the high content of growth factors in the platelets [26]. PRP has been shown to promote cell growth [27,28] and has been used in various tissue engineering applications in bone [29], [30], cartilage [31,32], skin [33,34], and in vivo applications [35]. These approaches exploit the release of chemo-attractive, angiogenic, proliferative, and putatively pro-regenerative growth factors from PRP, making it suitable as a cell delivery vehicle [36]. The difficulty of controlling the growth factor and cytokine release in PRP makes it necessary to combine it with other molecules, mainly polyethylene glycol (PEG) or chitosan, in order to regulate this [30,33,34,37,38,39]. Although PRP has shown its potency and capability in tissue regeneration, its experimental inconsistency and low reproducibility has meant that, in applications in which platelet-released growth factors are not crucial, PPP is generally used. Additionally, since PPP is cost-effective, suitable for bulk production, and easily translatable with minimal regulatory requirements for FDA approval, it is widely used in tissue regeneration [22,40,41,42,43].

In this context, our group engineered a human plasma-derived (PPP) bilayered skin substitute, with a final fibrin concentration of 1.2 mg/mL, containing primary human fibroblasts (hFBs) and keratinocytes (hKCs) from skin biopsies for different skin tissue engineering applications [44]. These applications include (1) treating burns and traumatic and surgical wounds in a large number of patients in Spain [15,45,46] (2) generating skin-humanized mouse models [47,48,49]; and (3) developing a complete system to 3D print this bilayered skin for clinical and commercial testing purposes. However, we found several persisting issues associated with the low final concentration of fibrin (1.2 mg/mL) used in the plasma derived-fibrin hydrogels when they are placed in transwell inserts for in vitro applications: (1) their height is reduced by 30% during the first 24 h and by 70% after 21 days in culture [17]; and (2) they suffer rapid degradation due to the skin cells present in the culture, limiting their lifespan (usually to 17 days) [15,44]. Several strategies have been proposed to overcome the limitations of plasma-derived fibrin-based hydrogels in skin tissue engineering, for example, combining fibrin (blood plasma-derived) with other molecules such as PEG or agarose polymers [40,41,42,50,51] or the use of highly concentrated commercial fibrinogen. In our experience, the use of commercial fibrinogen in organotypic skin cultures produced worse keratinocyte proliferative and differentiation behavior in comparison with PPP-hydrogel cultures. Additionally, by controlling the final fibrin concentration, it is possible to improve the mechanical performance of plasma hydrogels without the need to combine it with other molecules.

In this study, our objective was to increase the final fibrin concentration from 1.2 mg/mL to 2.4 mg/mL in human plasma-derived (PPP) fibrin hydrogels to overcome the aforementioned limitations. These numbers arise as two potentially optimal final fibrin concentrations due to the limited range of fibrinogen available in human plasma (2–4 mg/mL) [52,53]. The objective of the experiments was to determine the optimal final fibrin concentration for designing a functional skin dermo-epidermal equivalent for in vitro applications. In this way, a series of experiments that characterize the behavior of plasma-derived hydrogels (1.2 mg/mL and 2.4 mg/mL) in terms of gelation time, mechanical properties, contractile behavior, and biological performance were performed with the aim of improving skin culture lifespan.

## 2. Results and Discussion

### 2.1. Gelation Time and Kinetics

**Figure 1:**
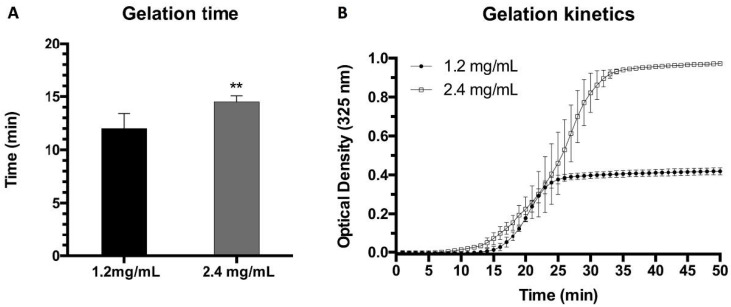
(**A**) Gelation times obtained using the flip-flop method for plasma hydrogels at 1.2 mg/mL and 2.4 mg/mL final fibrin concentrations (** *p* < 0.01); (**B**) Gelation kinetics obtained using UV spectroscopy and measured at 325 nm for plasma-derived hydrogels at 1.2 and 2.4 mg/mL final fibrin concentrations. OD: Optical density.

To determine gelation time and kinetics, two frequently used methods were followed: the flip-flop and optical absorbance (turbidimetry) methods. Hydrogel gelation time was first studied using the flip-flop method and the results for hydrogels containing final concentrations of 1.2 mg/mL and 2.4 mg/mL of fibrinogen showed a slight difference between the two types of gels (Figure 1A). The times obtained from the flip-flop method were manifestly shorter than those obtained by turbidimetric assays (Figure 1B), indicating that polymerization, as determined using the aforementioned method, was far from complete. The polymerization kinetics were analyzed using UV spectroscopy at 325 nm, as this wavelength provided the maximal O.D. difference between the nonpolymerized and polymerized estates of both types of gels (see Figure S1). This wavelength is similar to that reported for plasma-derived and commercial fibrinogen-derived fibrin hydrogels [17] and is in the range of those previously reported in the literature, i.e., between 300 nm and 550 nm, with a lower wavelength being unsuitable due to potential interference with proteins and DNA [32,54,55,56].

On the basis of different techniques, such as light scattering, tubidimetry, confocal and electron microscopy, and permeation analyses [57,58,59] there are several reports that demonstrate that, after an initial phase of nucleation and scaffold formation involving only 10–20% of the monomer concentration, the remainder of the gel formation mostly consists in the thickening of its fibers. Accordingly, the measured turbidity is directly related to the diameter of the fibers, and the OD plateau is reached when said diameter reaches its maximum value. Values around 130 nm have been reported using this technique [58]. The shape and gelation time of our turbidimetry curves and the fiber diameter of 170 nm obtained by SEM (see Section 2.3: Structural Characterization) are similar to those reported in the aforementioned studies.

### 2.2. Mechanical Characterization

**Figure 2:**
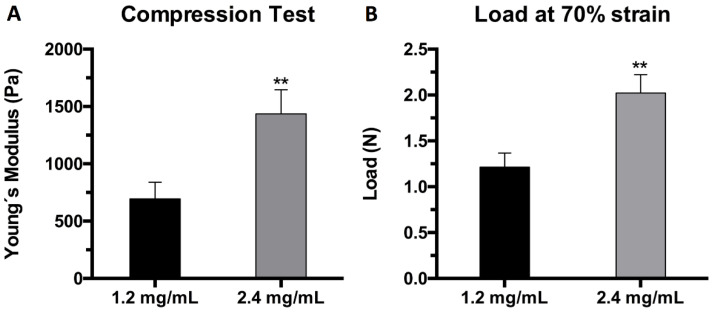
Mechanical characterization of plasma hydrogels: (**A**) elastic modulus (Pa) of 1.2 mg/mL (black) and 2.4 mg/mL (grey) samples at 20% strain (linear region); (**B**) load (N) at 70% strain of 1.2 mg/mL (black) and 2.4 mg/mL (grey) hydrogel samples (** *p* < 0.01).

Plasma hydrogels at both final fibrin concentrations (1.2 mg/mL and 2.4 mg/mL) were subjected to a uniaxial compressive test to assess their mechanical properties. The test was carried out in triplicate with samples of 5 mL final volume after a day of equilibration in PBS. The results were expressed in terms of the elastic modulus of compression or Young’s modulus (E) calculated from the stress vs. strain curve at 20% strain (linear region), and the maximum load withstood by the hydrogels when subjected to 70% strain. The results show that increasing the fibrin concentration from 1.2 mg/mL to 2.4 mg/mL indeed affected the mechanical properties by doubling the elastic moduli and the maximum load. The Young’s modulus value at 20% strain increased from 776 Pa to 1533 Pa, doubling the rigidity of the hydrogels (Figure 2A). Similarly, the maximum load at 70% strain increased from 1.24 N to 2.02 N when the final fibrin concentration was doubled from 1.2 mg/mL to 2.4 mg/mL (Figure 2B). These results are in agreement with those reported concerning the effect of fibrin and thrombin concentrations on the stiffness of fibrin hydrogels [60].

### 2.3. Structural Characterization

**Figure 3:**
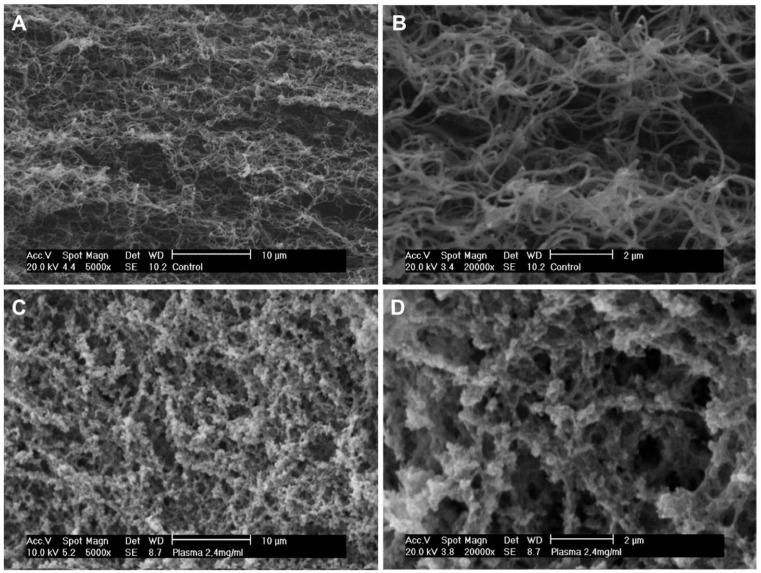
Representative images of plasma-derived hydrogels after 72 h of incubation at 37 °C under SEM with (**A**) 1.2 mg/mL fibrin concentration at 5000×, (**B**) 1.2 mg/mL fibrin concentration at 20,000×, (**C**) 2.4 mg/mL fibrin concentration at 5000×, and (**D**) 2.4 mg/mL fibrin concentration at 20,000×.

Plasma hydrogel internal morphology was assessed using scanning electron microscopy (SEM). The images obtained show clear differences in the structure and overall porosity when doubling the fibrin concentration (Figure 3). Hydrogels with a fibrin concentration of 1.2 mg/mL exhibited smooth and intertwined fibers with a measured average fiber size of 170 nm (Figure 3A,B). In the case of the 2.4 mg/mL hydrogels, the fibers tend to aggregate to form a conglomerate structure, in which porosity decreases and no fiber diameter could be measured (Figure 3C,D). Differences in the structural nature of the two concentrations are in agreement with the differences found in their mechanical properties (Figure 2). Hydrogels at a 1.2 mg/mL final fibrin concentration were found to be more porous and thus less mechanically stable.

### 2.4. hFB-Mediated Matrix Contraction, Proliferation, and Live/Dead Assays

**Figure 4:**
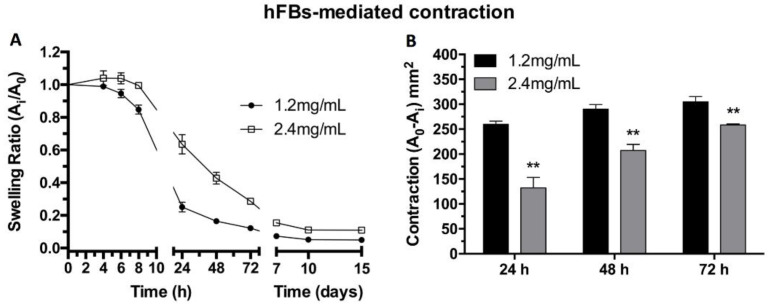
(**A**) Matrix contraction of free-floating hFBs-containing plasma-derived hydrogels at 1.2 mg/mL and 2.4 mg/mL final fibrin concentrations. (**B**) Quantification of the contraction area (mm^2^) at 24, 48, and 72 h of contraction kinetics shown in (**A**) A_0_ = 3.8 cm^2^ (** *p* < 0.01).

hFB behavior inside the matrices was studied both in terms of capability to contract the fibrin hydrogels and proliferation. The attachment of human primary fibroblasts to the fibrin matrix through the matrix RGD cell adhesion motifs was supported by the observation that the free-floating fibroblast-laden hydrogels tend to contract at a quicker pace than in the absence of cells. The capacity of human primary fibroblasts to contract the hydrogels was studied through the swelling ratio estimated as the change in surface area displayed by the hydrogels, which was measured through photographs (Figure 4). The results show that doubling the concentration of fibrin, and thus making hydrogels more mechanically stable (Figure 2), decreased the contraction of the hydrogels in the x-y plane (Figure 4A). Furthermore, the surface area change between both types of gel was calculated and statistically significant differences were found at 24, 58, and 72 h (Figure 4B), again evidencing the higher resilience of the more concentrated gels. The presence of fibroblasts in the free-floating hydrogels drastically augment their contractile behavior compared to similar but acellular plasma-derived fibrin gels [17].

**Figure 5:**
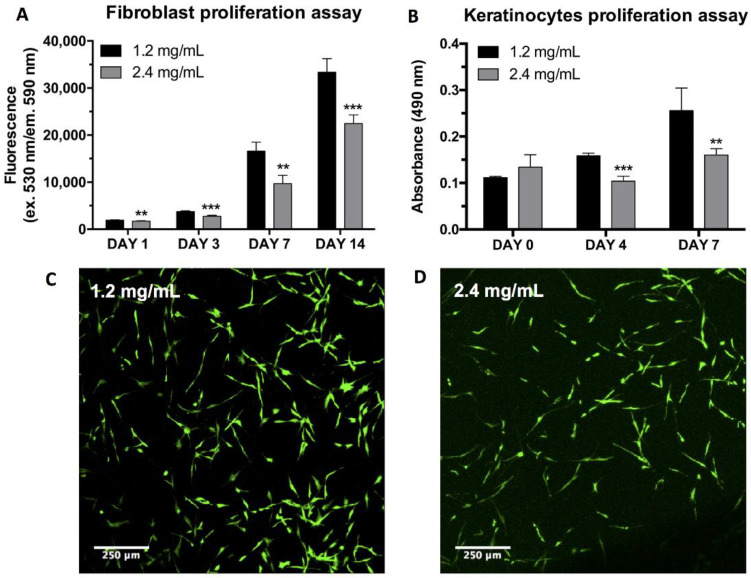
(**A**) Fibroblast proliferation inside the plasma hydrogels at 1.2 mg/mL and 2.4 mg/mL final fibrin concentrations measured through the Alamar Blue™ assay; (**B**) Proliferation of keratinocytes seeded on top of plasma hydrogels at 1.2 mg/mL and 2.4 mg/mL final fibrin concentrations at different timepoints, measured using the MTS assay. (** *p* < 0.01, *** *p* < 0.001); Fibroblast proliferation inside the plasma hydrogels at 1.2 mg/mL (**C**) and 2.4 mg/mL (**D**) final fibrin concentrations measured using the Live/Dead^®^ cytotoxicity assay after 48 h of culture at 37 °C.

The proliferation of human primary fibroblasts inside the two different plasma hydrogels was studied using the Alamar Blue™ assay. The results evidence various differences between the two hydrogel systems. hFBs embedded in gels with a final fibrin concentration of 2.4 mg/mL exhibited a lagging behavior in terms of cell proliferation (Figure 5A). The metabolic activity of fibroblasts incubated in 1.2 mg/mL plasma hydrogels was always higher than that found in the 2.4 mg/mL hydrogels in the temporal interval studied.

These results are somewhat surprising since, for stiffnesses similar to those found in our gels (Figure 2) using the microposts experimental approach, it was shown [61] that the NIH 3T3 fibroblast cell spread area, focal adhesion formation, and cell proliferation rate increased monotonically with substrate rigidity. Similar results have been repeatedly reported for different cell types using this and other experimental setups (e.g., [62]). However, cell–matrix interactions are probably very different and more complex in a 3D fibrin scaffold than on a micropost.

Therefore, this behavior is in agreement with previous reports on the proliferation of cells with increasing fibrin concentrations. This is due to the more compact matrix delaying the remodeling by the fibroblasts [60,63].

Further analysis of the effect of fibrin concentration was conducted using the Live/Dead assay. This assay served to prove thatwhether hFBs can be embedded inside the plasma hydrogels and can survive by spreading and dividing inside the gels. The results show that fibroblast morphology is maintained regardless of the final fibrin concentration used; however, as previously demonstrated by the Alamar Blue™ assay, proliferation was found to be lower for the 2.4 mg/mLml fibrin hydrogels (Figure 5C,D).

### 2.5. hKC Proliferation Assay

hKCs’ ability to attach and proliferate on top of the hydrogels was studied using the MTS assay. The results show that the initial proliferation was very similar in both types of hydrogel. However, after 4 and 7 days of culturing, hKCs continued to proliferate at a stable rate in the 1.2 mg/mL fibrin hydrogels, while their proliferation stagnated in the case of the more concentrated plasma hydrogels (Figure 5B). Contrary to what is reported in the literature [62,64], our results demonstrate that stiffer surfaces slow the keratinocyte cells’ rate of proliferation in our system This might be due to both the stiffness and the porosity and topography of the hydrogels affecting proliferation. The results shown in Figure 4 and Figure 5 indicate that it is necessary to reach a compromise between the mechanical stability of fibrin gels and cell proliferation.

### 2.6. Contraction of Organotypic Skin Construct (Z Axis)

**Figure 6:**
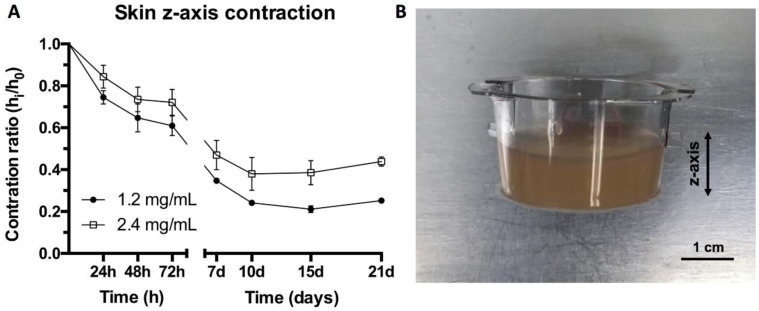
(**A**) Contraction ratio of organotypic skin constructs with final fibrin concentrations of 1.2 mg/mL and 2.4 mg/mL. (**B**) Plasma hydrogel cultured in transwell plate.

The gel contraction results shown in Figure 4 were obtained in order to characterize gel behavior using free-floating wall-detached fibrin gels. However, when performing 3D organotypic skin cultures, the gels remained attached to the walls of the transwells and did not contract on the x-y axes but on the z axis. Therefore, to study the usefulness of the two types of gel, it was necessary to measure their contraction in the z axis. This contraction was evaluated through photographs that were later analyzed using the ImageJ software. The height of the hydrogels was measured while the differentiation and maturation of the skin was taking place. The results show a decrease in the contraction for the 2.4 mg/mL fibrin hydrogels. The differences in height were around 10–12% for the first 7 days, while after 10 days, this difference increased to 14% and further increased to around 18% at 15 and 21 days (Figure 6A).

These results are in qualitative agreement with those shown in Figure 4, which were obtained with free-floating gels, as the higher fibrin concentration gels contract less in the z axis. However, their long-term contraction clearly diminished under organotypic culture conditions. We previously reported similar results with regard to the lower z-axis contraction of 1.2 mg/mL fibrin gels under culture conditions compared to free-floating gels. We attributed this, at least in part, to the presence of 10% FCS in the culture medium, which prevented or decreased the plasma proteins trapped in the gel from escaping [17]. This together with their greater mechanical stability explain the lower shrinkage of the 2.4 mg/mL gels, which were obtained through the polymerization of a more concentrated plasma.

### 2.7. Study of Organotypic Skin Cultures

In order to analyze the biological behavior of the two different matrices, the structure and viability of 3D organotypic skin constructs were studied for both conditions. For this purpose, the skin cultures were subjected to histological analyses throughout the period of epidermal differentiation in the culture phase at the air–liquid interface. Following our protocol, it took approximately 15 days to obtain a well-differentiated epidermis.

**Figure 7:**
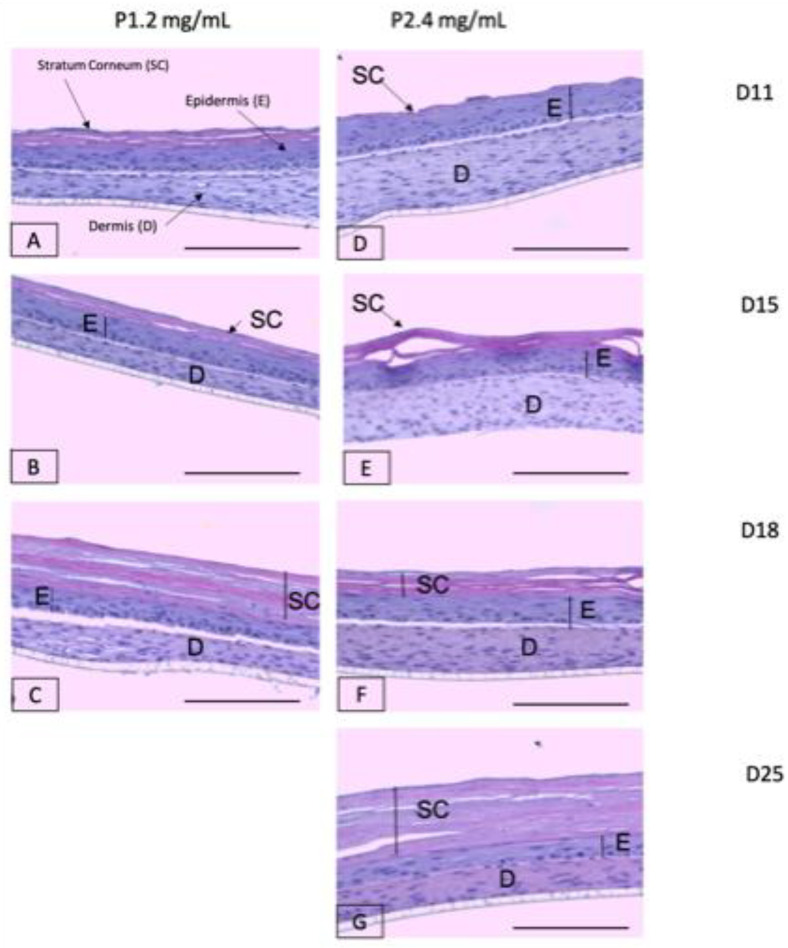
Hematoxylin and eosin (H&E) staining of organotypic skin cultures with matrices of two different fibrin concentrations: 1.2 mg/mL (**A**–**C**) and 2.4 mg/mL (**D**–**G**) at four different timepoints (11, 15, 18, and 25 days after placing them at the air–liquid interface for epidermal differentiation). SC: Stratum Corneum; E: epidermis; D: dermis. Scale bar: 500 µm.

One of the objectives of this study was to determine the experimental conditions that extend the lifespan of these cultures in order to increase their usefulness. As can be seen in Figure 7, which shows the three experiments, the two types of gels exhibited cultures with a well-stratified and cornified epidermis from the histological point of view. For the 1.2 mg/mL fibrin gels (Figure 7A–C), it was observed that the height of the dermal compartment (fibrin gel with fibroblasts) decreased over time, i.e., it was very thin at day 18, while at day 25, the culture disappeared. In the case of the 2.4 mg/mL fibrin gels (Figure 7D–G), the dermal compartment also reduced in height over time, but this reduction was slower than for the 1.2 mg/mL gels. As can be seen by comparing Figure 7A,D, Figure 7B,E, Figure 7C,F, the dermis of the 2.4 mg/mL fibrin cultures was always thicker than that of the 1.2 mg/mL fibrin cultures at any timepoint. As a consequence of this slowdown in matrix degradation, skin constructs with 2.4 mg/mL matrices lasted at least up to day 25, with their integrity intact.

**Figure 8:**
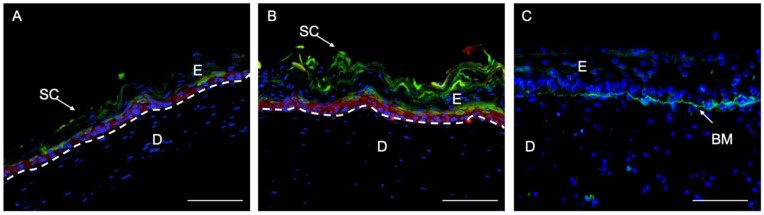
Immunofluorescence (IF) analysis of organotypic skin cultures using epidermis-specific markers: keratin 5 (K5), characteristic of basal epidermal cells (red staining, in (**A**,**B**)); keratin 10 (K10), characteristic of suprabasal epidermal cells (green staining, in (**A**,**B**)); filaggrin, characteristic of the stratum granulosum, the last compartment containing living cells before dead and cornified cells (green staining, in (**A**,**B**)) and collagen IV (Col IV), component of the lamina densa of basememnt membrane (green staining in (**C**)). (**A**) Organotypic skin cultures (1.2 mg/mL); (**B**) organotypic skin cultures (2.4 mg/mL) after 15 days of differentiation at the air-liquid interface; (**C**) Organotypic skin culture (2.4 mg/mL) after 25 days of differentiation at the air-liquid interface. SC: Stratum Corneum; E: epidermis; D: dermis; BM: Basement Membrane. Dotted white line indicates the dermo-epidermal junction (basal membrane). Scale bar: 500 µm.

**Figure 9:**
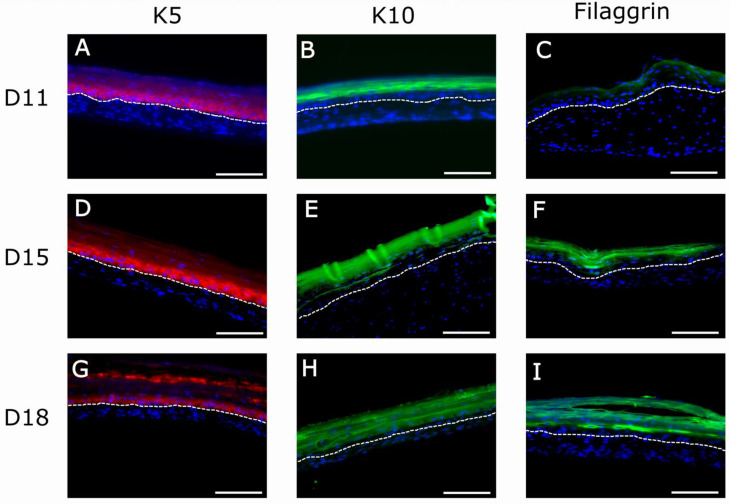
Immunofluorescence (IF) analysis of organotypic skin cultures (1.2 mg/mL) using epidermis-specific markers: keratin 5 (K5), characteristic of basal epidermal cells (red staining in (**A**,**D**,**G**)); keratin 10 (K10), characteristic of suprabasal epidermal cells (green staining in (**B**,**E**,**H**)); and filaggrin, characteristic of the stratum granulosum, the last compartment containing living cells before dead and cornified cells (green staining in (**C**,**F**,**I**)) at three timepoints of differentiation at the air–liquid interface (11, 15, and 18 days). Blue spots in (**A**–**I**) correspond to cell nuclei stained with DAPI. The bright fluorescent lines in (**E**) are an artifact caused by folds present in the sample during histological processing. Dotted white line indicates the dermo-epidermal junction (basal membrane). Scale bar: 200 µm.

**Figure 10:**
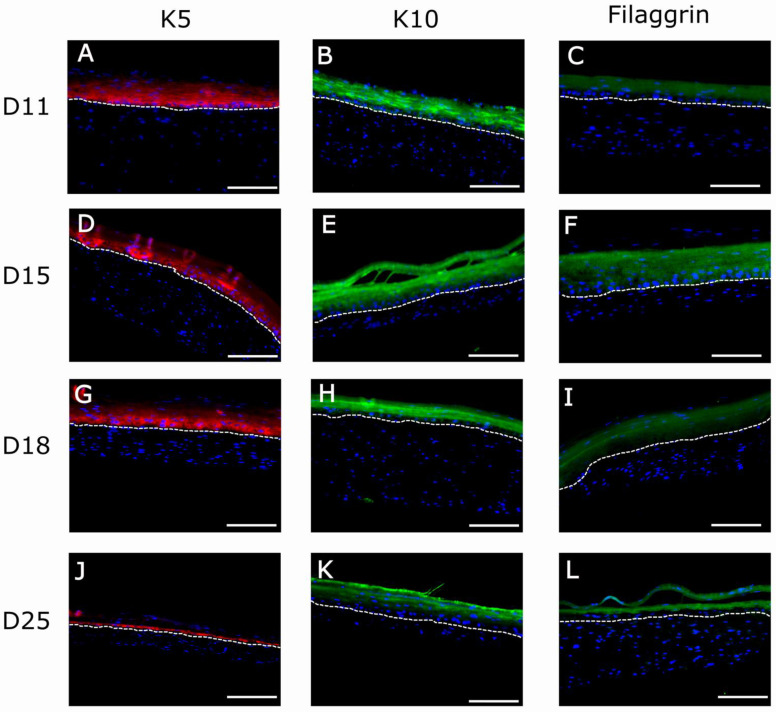
Immunofluorescence (IF) analysis of organotypic skin cultures (2.4 mg/mL) of human-specific epithelial markers: K5 (**A**,**D**,**G**,**J**), K10 (**B**,**E**,**H**,**K**), and Filaggrin (**C**,**F**,**I**,**L**) at four different timepoints of development (11, 15, 18, and 25 days). Dotted white line indicates the dermo-epidermal junction (basal membrane). The bright fluorescent lines in (**D**) are an artifact caused by folds present in the sample during histological processing. Scale bar: 200 µm.

To confirm the H&E results of Figure 7, we performed immunofluorescence experiments using well-known markers of epidermal differentiation: collagen IV for lamina densa in the basement membrane, keratin K5 for the basal layer of the epidermis, keratin K10 for the suprabasal epidermal layers, and filaggrin for cells of the very suprabasal stratum granulosum which, upon death and cornification, give rise to the stratum corneum (Figure 8). Although at day 11 the epidermis of both types of matrices seemed well differentiated according to histological criteria (Figure 7A,D), the filaggrin immunostaining analysis demonstrated poor (Figure 9C) or a lack (Figure 10C) of terminal differentiation in the 1.2 mg/mL and 2.4 mg/mL fibrin gels, respectively. This could indicate a slight delay in the differentiation of the epidermis that developed in the 2.4 mg/mL fibrin gels. According to this criterium, a fully differentiated epidermis, characterized by strong filaggrin staining, was only present at day 15 in both types of matrices.

For both fibrin concentrations, at longer maturation times, the stratum corneum increased in height (compare Figure 7A,C and Figure 7D,G, respectively) whereas the suprabasal compartment decreased in dimension, and the basal cell compartment remained the same for all conditions and times.

This observation was confirmed using the immunofluorescence analysis of epithelial markers (Figure 8) in organotypic skin cultures (1.2 mg/mL and 2.4 mg/mL) after 15 days of differentiation at the air–liquid interface. At day 15, all epithelial markers reached their maximal expression, as can be seen by the intensity of their immunofluorescence (Figure 9D–F and Figure 10D–F). This is the point at which the epidermis could be considered best differentiated. For longer culture times, all marker expressions seemed to decrease with time (Figure 9G–I and Figure 10J–L). However, expression of collagen IV in basement membrane is barely detectable from day 18 (data not shown), being more prominent at day 25 (Figure 8C). Thereby, collagen IV and collagen VI [65,66,67,68] are typical markers of skin basement membrane which are expressed in vitro after 3 weeks [69]. Their expression is more evident in human skin in vivo when grafted to immunodeficient mice eight weeks post-grafting [9,70]. This observation supports the need for improving the mechanical properties of organotypic skin cultures to extend culture times, as collagen IV expression was only noticeable at day 25 in 2.4 mg/mL organotypic skin cultures.

Epidermal thickening, caused solely by stratum corneum increase, demonstrated homeostasis unbalance in the epidermis. Homeostasis is the maintenance of the mass and the integrity by balanced cell gain and loss, as occurs in vivo. However, this balance is not achieved in vitro, as the stratum corneum increases in thickness, compromising the rest of the epidermis layers. This phenomenon has been reported in the literature [69,71] and is mainly attributed to unbalanced epidermal homeostasis. As the epidermis continuously differentiates and natural desquamation does not occur in static in vitro cultures, there is an accumulation of keratinized cells layers. Therefore, to demonstrate the effect that the shedding of the stratum corneum has on the maintenance of proper epidermal homeostasis [69], dynamic culture systems able to dislodge keratinized sheets need to be developed. On the other hand, this could also be a consequence of the culture conditions we used to promote epidermal differentiation, which imply keeping the dermo-epidermal cultures for 3 weeks at the air–liquid interface under low-serum conditions. It is possible that these conditions led to an imbalance between keratinocyte proliferation and differentiation, favoring the latter. Thus, different culture media conditions and compositions should be analyzed to find a balance between these two processes.

## 3. Materials and Methods

### 3.1. Gelation Time and Kinetics

#### 3.1.1. Plasma-Derived Fibrin Hydrogel Preparation

Frozen human plasmas with a low platelet content were obtained from a blood bank in Spain (Banco de Sangre del Centro Comunitario de Transfusión del Principado de Asturias (CCST)) and treated according to the standards of the American Association of Blood Banks [72]. The experiments described in this article were performed using plasma of known fibrinogen concentrations from different batches to ensure reproducibility. Plasma-derived hydrogels were prepared following the protocol previously described by Meana et al. [44]. Briefly, calculations were performed to attain the volume of plasma needed to obtain hydrogels with a final fibrinogen concentration of both 1.2 mg/mL and 2.4 mg/mL together with the antifibrinolytic agent tranexamic acid (AmchaFibrin 500 mg, Meda Pharma SL, Madrid, Spain) at a final concentration of 0.008% *w*/*v*. Finally, in order to trigger the coagulation cascade, sterile filtered calcium chloride (CaCl_2_ 1%, Sigma Aldrich, Burlington, VT, USA) was added to a final concentration of 0.08% (*w*/*v*) for the 1.2 mg/mL hydrogels and 0.16% *w*/*v* for the hydrogels with a 2.4 mg/mL final fibrin concentration. The mixture was adjusted to the final desired volume by adding the corresponding volume of sodium chloride (NaCl 0.9% (*w*/*v*), Sigma Aldrich, Burlington, VT, USA). The final mixture was introduced into the appropriate recipient (glass vial, culture plate, and falcon tube) and incubated for 1 h at 37 °C in an atmosphere containing 5% CO_2_ and at a 40% relative humidity for their gelation.

#### 3.1.2. Gelation Time and Kinetics

The differences in gelation time of the 1.2 and 2.4 mg/mL human plasma-derived hydrogels were determined using the flip-flop method [17]. Glass vials containing the hydrogel mixtures were tilted every 30 s until no liquid was left in the vial and the hydrogel was stuck to the bottom; this moment was denoted as the final gelation time. Further characterization of the gelation process was achieved through the study of the gelation kinetics with UV turbidimetry assays. The gelation kinetics were monitored using a Synergy™ HTX Multi-Mode Microplate Reader (Winooski, VT, USA). Hydrogel solutions were placed into a solid glass 96-well plate (6.4 mm diameter; 0.32 cm^2^ area) and quickly transferred to the microplate reader for measurement. A glass system plate was used to ensure the proper gelation of the plasma hydrogels in the absence of hFBs. Once the optimal UV-wavelength was assessed, as previously described [17] (see Supplementary Figure S1), gelation experiments were performed by incubating the hydrogel solutions at 37 °C for 1 h, with absorbance reading intervals of 30 s to accurately characterize their gelation kinetics.

#### 3.1.3. Mechanical Characterization: Compression Test

Both final fibrin concentration hydrogel types were subjected to a compression test using an electromechanical system Instrom 3366 to compare their mechanical properties. The compression rate was set at 2 mm/s. Hydrogels were prepared in 25 mm diameter glass vials, detached, and left to incubate at 37 °C in PBS (Sigma Aldrich, Burlington, VT, USA) for 24 h prior to testing in order to reach equilibrium. The final volume of the samples was 5 mL, giving an initial height of 1 cm. The results were expressed in terms of the stress/strain curve and the compressive modulus of elasticity or Young’s modulus (E) at 20% strain. Furthermore, the maximum load withstood by the hydrogel at 70% strain was recorded.

#### 3.1.4. Structural Characterization (SEM)

Human plasma-derived hydrogels with fibrin concentrations of 1.2 mg/mL and 2.4 mg/mL were structurally characterized using scanning electron microscopy (SEM). Plasma hydrogels with a final volume of 3 mL were prepared and detached as described above and then transferred to a series of ethanol dilutions (20%, 40%, 60%, 80%, and 100%) for sample dehydration for their critical CO2 point drying [73]. A supercritical CO_2_ reactor (Thar R100W) was used under the conditions stated in previous work [17]. Images were obtained at different magnifications (5000×, 10,000×, and 20,000×) using a Philips XL30 scanning electron microscope.

#### 3.1.5. Primary hKCs and hFBs Culture

Primary cells (hFBs and hKCs) from skin biopsies of healthy donors were obtained from collections of biological samples of human origin; these samples are registered in the ‘Registro Nacional de Biobancos para Investigación Biomédica del Instituto de Salud Carlos III’. In this work, we used cells from different donors. hKCs were cultured according to the previously described methods [74] and then modified by our laboratory [44,75]. The growing media for hKCs was a 3:1 mixture of Dulbecco’s modified Eagle medium (DMEM) (GIBCO™-BRL), HAM’S F12 (GIBCO™-BRL), and 1% penicillin/streptomycin (P/S) (hKC medium), containing 10% of fetal calf serum (FCS), 0.1 nM choleric toxin, 2 nM T3, 5 mg mL^−1^ insulin, 0.4 mg mL^−1^ hydrocortisone, and 10 ng mL^−1^ EGF (Sigma, St Louis, MO, USA). hFBs were cultured in Dulbecco’s modified Eagle’s medium (DMEM, Biochrom KG, Cambridge, UK) containing 10% FBS and 1% (P/S).

#### 3.1.6. hFBs-Mediated Contraction

Human plasma hydrogels at fibrin concentrations of 1.2 mg/mL and 2.4 mg/mL containing hFBs at a cell density of 80,000 cells/mL of hydrogel were prepared as described [17]. After incubation for 1 h at 37 °C in glass vials, hydrogels were detached with DMEM supplemented with 10% FBS and 1% P/S pre-warmed at 37 °C and incubated free-floating in the same culture medium at 37 °C, 5% CO_2_. The culture medium was changed every 2 days. Images of the hydrogel at each timepoint (0 h, 4 h, 6 h, 8 h, 24 h, 48 h, 72 h, 7 days, 10 days, and 15 days after detaching) were obtained using a digital camera for image analysis and area measurement. The swelling area ratio was calculated with the following equation:

(1) $$SR_{A}=\frac{A_{T}}{A_{0}}$$

where *SR_A_* is the swelling ratio in area, *A_T_* is the surface area at each timepoint, and *A*_0_ is the initial area of the hydrogel.

#### 3.1.7. hFB Proliferation and Life/Death Assay

hFB proliferation inside the plasma hydrogels was studied using the Alamar Blue™ assay, following the manufacturer’s instructions. Briefly, 100 µL of hydrogels were prepared including hFBs at a density of 80,000 cells/mL of gel in Costar Black 96-well plates with 6 replicates per condition. Hydrogels with final fibrin concentrations of 1.2 mg/mL and 2.4 mg/mL at different timepoints (1 day, 3 days, 7 days, and 14 days) were incubated with 100 µL of Alamar Blue working solution for 3 h at 37 °C and 5% CO_2_. After incubation, the supernatants were transferred to a 96-well plate in order to measure fluorescence in the Synergy™ HTX Multi-Mode Microplate Reader (excitation/emission: 570 nm/600 nm).

hFBs viability inside both types of hydrogels was further characterized with the Live/Dead Viability/Cytotoxicity kit (Thermofisher, Frederick, MA, USA). Hydrogels were prepared in m-slide 8-well glass bottom plates (Ibidi GmbH, Grafelfing, Germany). Thereafter, 100 µL of plasma with 160,000 cells/mL were studied. After gelation, culture medium containing 10% FBS and 1% P/S was added on top and the hydrogels were incubated for 48 h. Live/Dead^®^ staining was performed by adding calcein AM and ethidium homodimer at final concentrations of 0.5 μL/mL and 2 µm/mL in PBS, respectively. After 30 min at 37 °C, any excess reagent was washed with PBS and the viability of hFBs inside the hydrogels was visualized using confocal microscopy.

#### 3.1.8. Keratinocyte Proliferation Assay (MTS)

The proliferation capacity of hKCs was assessed using an MTS assay (Abcam, Cambridge, UK), following the manufacturer’s instructions. Hydrogel samples were prepared in 96-well plates in the absence of hFBs. To allow for keratinocyte proliferation in the absence of fibroblasts, conditioned culture media CnT-57 (CELLnTEC Advanced Cell Systems Bern, AG, Bern, Switzerland) was used during the study. Plasma hydrogel final volume was 100 µL and gelation was left to proceed for 2 h to ensure that gels correctly gelified. After this step, 100 µL of conditioned medium was added for 4 h in order to eliminate the excess calcium inside the hydrogels. Finally, hKCs at a cell density of 100,000 cells/mL in conditioned culture medium were seeded on top of the constructs and left undisturbed for 48 h at 37 °C, 5% CO_2_, and 40% relative humidity for cell attachment. After this, the medium was changed every 2 days and samples were taken at 1, 4, and 7 days. The medium was removed from each well, and 100 µL of MTS working solution was added. The plate was left for 2 h at 37 °C and 5% CO_2_ before the reduced medium was transferred to a new clean plate. The plate reduced media O.D. was measured at 490 nm in a Synergy™ HTX Multi-Mode Microplate Reader.

#### 3.1.9. Contraction of Plasma-Derived Fibrin Hydrogels in the Z Axis under In Vitro Skin Culture Conditions

Plasma hydrogels were prepared inside transwells (Fisher Scientific, Frederick, MA, USA) in order to generate dermo-epidermal skin constructs in vitro following the protocol previously reported by Meana et al. [44]. hFBs were incorporated at a 20,000 cells/mL and hydrogels were incubated for 1 h. Afterwards, fibroblast culture media was added and cultured for 24 h at 37 °C, 5% CO_2_, and 40% relative humidity. hKCs were seeded on top of the plasma hydrogels at a density of 1.5 × 10^6^ cells using keratinocyte culture media. Keratinocytes were left to sink and attach to the hydrogel surfaces for 48 h. Transwells were transferred to deepwell plates (Fisher Scientific, Frederick, MA, USA) to allow keratinocyte differentiation at the air–liquid interface. Photographs were taken of the profile of the transwells to measure the height of the hydrogels during the differentiation process. The defined timepoints were 0 h, 24 h, 48 h, 72 h, 7 days, 10 days, 15 days, and 21 days. Images were analyzed using the ImageJ software (Wayne Rasband, NIH).

### 3.2. Organotypic Skin Cultures

#### 3.2.1. Study of Organotypic Skin Cultures at Different Timepoints

The effect of plasma concentration on organotypic skin was studied at different timepoints. Dermo-epidermal cultures at final fibrin concentrations of 1.2 and 2.4 mg/mL were prepared and cultured following the methodology described above (Section 3.1.9). Skin differentiation and maturation was stopped at different timepoints (days 11, 15, 18, and 25, with time zero being when the organotypic skin culture is moved to the air–liquid interface) to analyze the skin structure. At each timepoint, a dermo-epidermal equivalent of each fibrin concentration was fixed in a formalin-free tissue fixer (Sigma Aldrich, Burlington, VT, USA) for 24 h. After this, they were paraffin-embedded for histological and immunofluorescence analyses.

#### 3.2.2. Histological and Immunofluorescence Analyses

For the structural analyses, 5 µm thick tissue sections were stained with hematoxylin and eosin (H/E) using standard protocols [76]. For the immunofluorescence analysis, 5 µm thick tissue sections were analyzed using primary specific antibodies against well-known skin markers: antihuman-vimentin (1:100, monoclonal MA5-11883, Thermofisher, Frederick, MA, USA to distinguish hFB), antikeratin 5 (1:250, monoclonal MA5-12596, Thermofisher, Frederick, MA, USA; to label hKC of the proliferative basal layer), antikeratin 10 (1:400, monoclonal MA5-13705, Thermofisher, Frederick, MA, USA; to label suprabasal keratinocytes), antihuman filaggrin (1:100, monoclonal MA5-13440, Thermofisher, Frederick, MA, USA; to label the epidermal granular layer) and anti-collagen IV (1:100, monoclonal 14-9871-82, eBioscience, San Diego CA, USA). Samples were incubated for 5 min with DAPI for nuclei staining and coverslipped using DPX (06522, Sigma Aldrich, Burlington, VT, USA). Image acquisition was performed using an inverted microscope Leica DMi8 and an objective HC PL Fluotar 20×.

### 3.3. Statistical Analysis

Statistical analysis was performed using the IBM-SPPS (IBM, Armonk, NY, USA) software. All data averages and standard deviations were subjected to paired Student’s *t*-test [77] in order to determine the potential statistical difference significance in the results. Three levels of significance were assessed: * *p* < 0.05; ** *p* < 0.01; *** *p* < 0.001.

## 4. Conclusions

In this study, we assessed the influence of fibrin concentration on the properties of fibrin matrices designed for organotypic skin cultures. For this, the mechanical, structural, and biological behaviors of matrices containing 1.2 mg/mL and 2.4 mg/mL of fibrin, obtained through the polymerization of human plasma, were extensively characterized. We chose this range of concentrations because, on the one hand, at lower concentrations, the hydrogels are too soft and, on the other hand, it is difficult to obtain matrices at higher fibrin concentrations from plasma.

Although both types of matrices gave rise to cultures with a well differentiated epidermis after 15 days of culture at the air–liquid interface, the 1.2 mg/mL hydrogels demonstrated poorer mechanical properties and cell-mediated contraction rates. This resulted in a maximal lifespan of 18 days, which limits the usefulness of these cultures. Contrarily, they displayed a somewhat better performance in terms of human primary fibroblast proliferation and keratinocytes proliferation and differentiation. Organotypic cultures using the 2.4 mg/mL hydrogels exhibited better mechanical properties of the dermal compartment. This allowed for longer culture times (25 days), potentially making them more useful.

However, in order to effectively extend the lifespan of skin organotypic cultures, it is necessary to find new culture conditions that avoid excessive formation of the stratum corneum and allow for a better balance between proliferation and differentiation of the epidermal compartment. The structural and functional integrity of the epidermal layer of these types of skin organotypic culture will be also assessed with transepidermal water loss (TWEL) and transepithelial electrical resistance (TEER) combined with in vitro skin permeation/penetration studies. These systems will allow the generation of more reliable testing platforms for pharmaceutical products and cosmetics, and future work will elucidate their potential.

## Data Availability

Due to the size of the raw files, datasets are only available upon request.

## Supplementary Materials

The following is available online at https://www.mdpi.com/article/10.3390/ijms22136746/s1, Figure S1: UV scan from 200 nm to 800 nm of (A) human plasma at 1.2 mg/mL (A) and at 2.4 mg/mL (B) of fibrinogen concentration before (empty circles) and after (filled circles) polymerization.
