# Supplementary material for: Effect of Fibrin Concentration on the In Vitro Production of Dermo-Epidermal Equivalents
Source: Int J Mol Sci. 2021 Jun 23;22(13):6746. doi: 10.3390/ijms22136746 (PMC8269027; doi:10.3390/ijms22136746)
Supplement: Supplementary file 1 [file ijms-22-06746-s001.zip › ijms-1156827-supplementary.pdf]

Supplementary

1. Turbidimetry study

These absorption spectrometry results showed that, in the range between 325 nm to 500 nm, both human plasma at 1.2mg/ml (Figure S1-A) and at 2.4 mg/ml (FigureS1- B) present important absorption differences between their sol and gel states. In view of these results, a wavelength of 325 nm was selected to follow by turbidimetry the gelation dynamics experiments of Figure 1B.

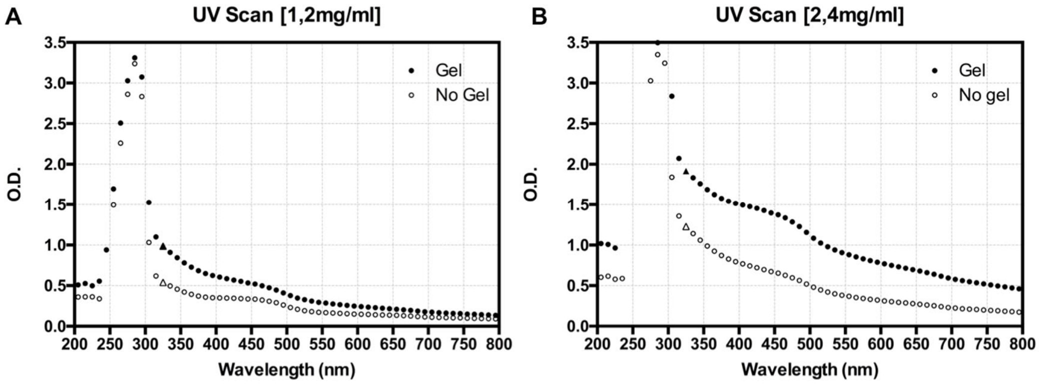

Figure S1. UV scan from 200 nm to 800 nm of (A) human plasma at 1.2mg/ml (A) and at 2.4 mg/ml (B) of fibrinogen concentration before (empty circles) and after (filled circles) polymerization.
